# Supplementary material for: Saliva Testing Is Accurate for Early-Stage and Presymptomatic COVID-19
Source: Microbiol Spectr. 2021 Jul 14;9(1):10.1128/spectrum.00086-21. doi: 10.1128/spectrum.00086-21 (PMC8552797; doi:10.1128/spectrum.00086-21)
Supplement: SUPPLEMENTAL FILE 1 — Supplemental material. Download SPECTRUM00086-21_Supp_1_seq7.pdf, PDF file, 0.4 MB [file spectrum00086-21_supp_1_seq7.pdf]

Supplemental Materials

Saliva testing is accurate for early-stage and presymptomatic COVID-19

AJ Johnson et al.

Table of Contents:

Supplemental Figure 1: Results from N2 primers agreement with N1 primers. 2

Supplemental Figure 2: Relative viral load results. 3

Supplemental Figure 3: Evaluation of Cohort 1B symptoms, cycle thresholds, and test platform. 4

Supplemental Table 1: Contingency comparisons for Cohort 1A. 5

Supplemental Table 2: Contingency comparisons for Cohort 2. 5

Supplemental Table 3: Contingency comparisons for Cohort 1B. 5

Supplemental Figure 4: Saliva and nasal collection devices. 6

Clinical Quality Assurance Data. 7

Supplemental Table 4: Contingency comparison of CLIA-LDT and Cepheid. 7

Supplemental Table 5: Contingency comparison of CLIA-LDT and Diasorin. 7

References. 7

Supplemental Figure 1: Results from N2 primers agreement with N1 primers.

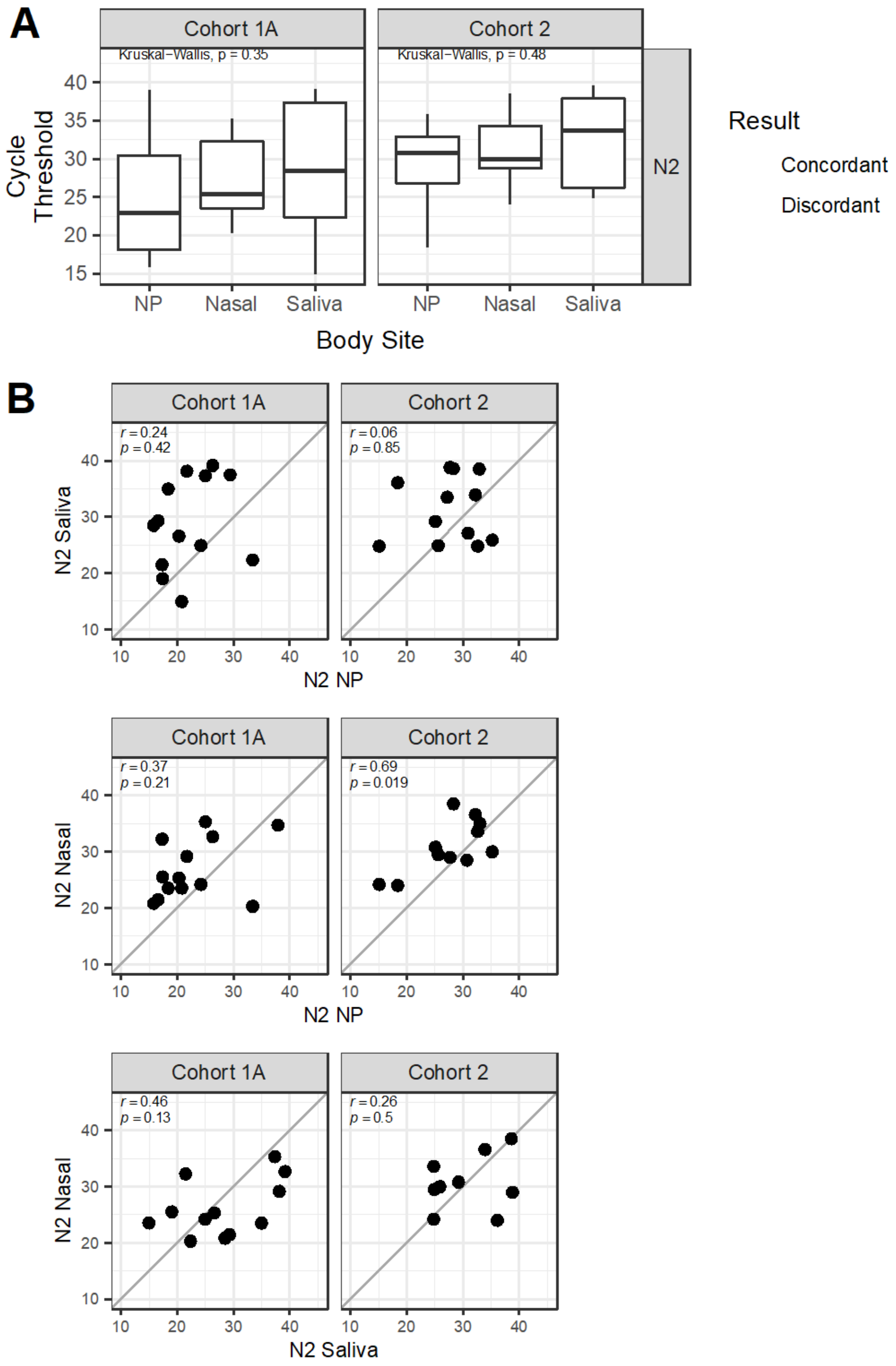

A) Box plots showing cycle threshold (Ct) values for Cohort 1A (n = 41) and 2 (n = 18) from the CLIA-LDT assay) for nasopharyngeal samples (NP), nasal samples, and saliva samples using N2 primers. Black circles are samples with concordance between nasal/saliva samples and NP samples (positive/positive or negative/negative). Grey squares show discordance between nasal/saliva samples and NP samples (positive/negative). Groups are compared using Kruskal-Wallis test by ranks. B) Scatter plots of Ct values from N2 primers for concordant samples from different sample types: saliva v. NP; nasal v. NP; nasal v. saliva. Correlation was using Pearson’s correlation. The correlation coefficient (r) and p-value for each comparison are indicated on each plot.

Supplemental Figure 2: Relative viral load results.

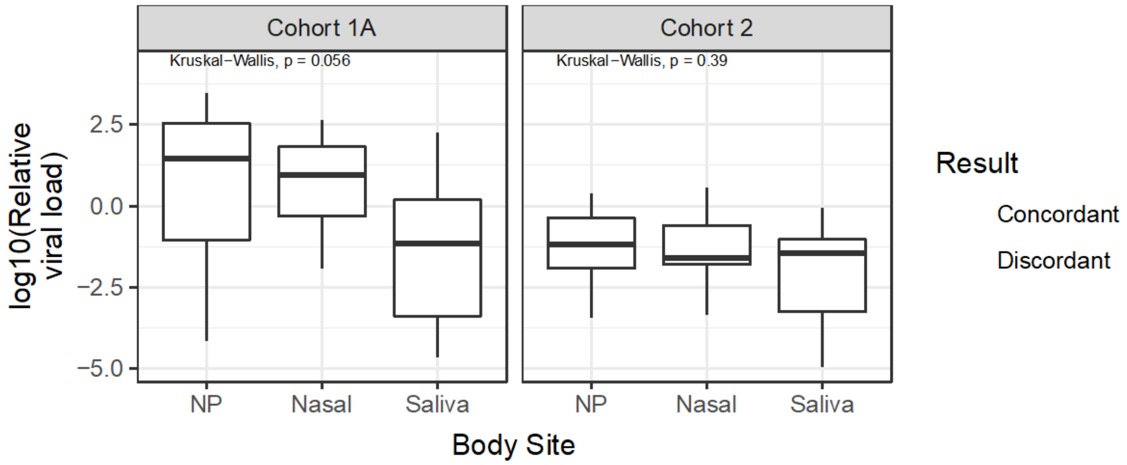

A) Box plots showing calculated relative viral load values for Cohort 1A (n = 41) and 2 (n = 18) for nasopharyngeal samples (NP), nasal samples, and saliva samples using N2 primers. Black circles are samples with concordance between nasal/saliva samples and NP samples (positive/positive or negative/negative). Grey squares show discordance between nasal/saliva samples and NP samples (positive/negative). Groups are compared using Kruskal-Wallis test by ranks.

Supplemental Figure 3: Evaluation of Cohort 1B symptoms, cycle thresholds, and test platform.

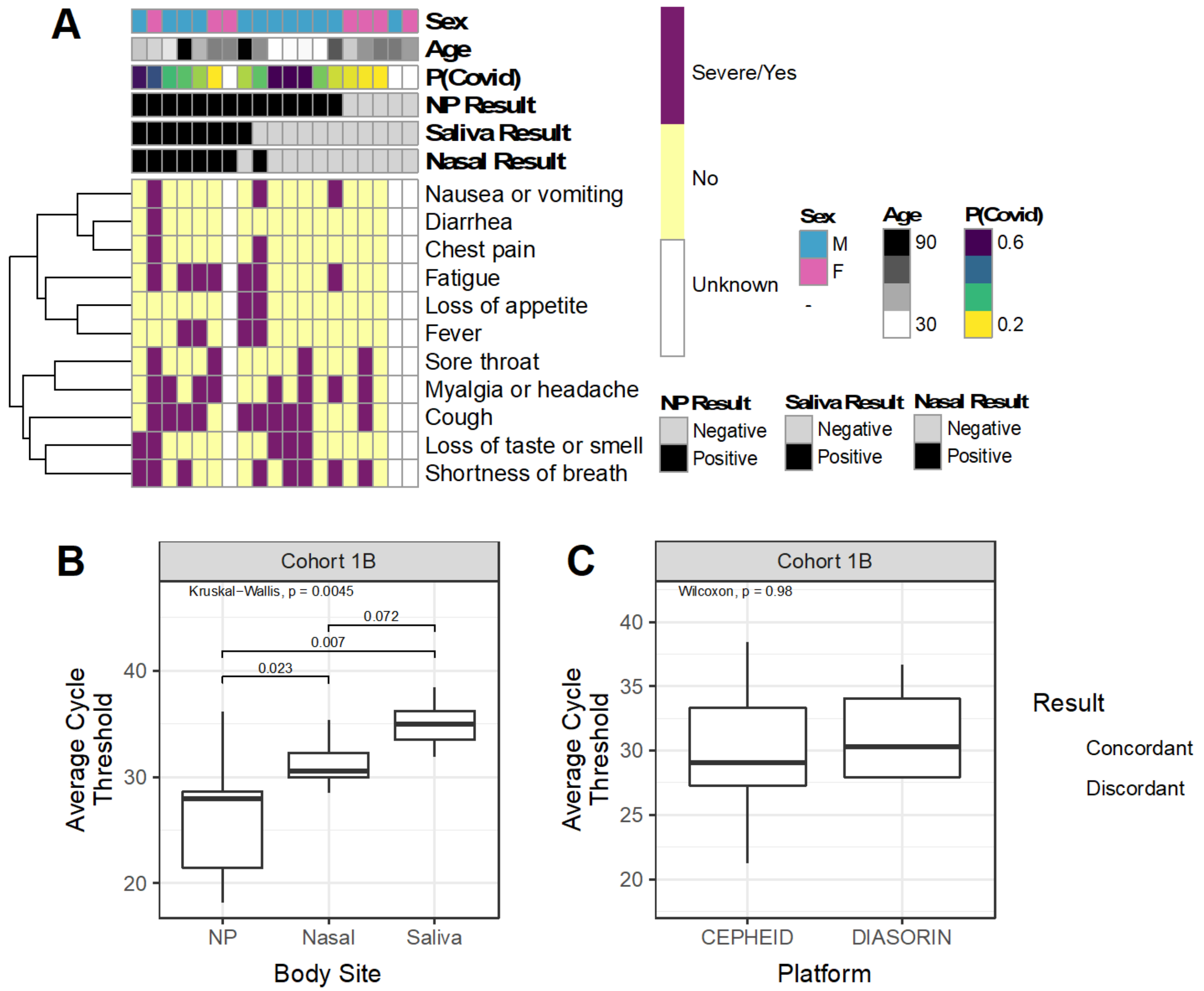

A) Heat map columns are participants and rows are symptoms. Symptom presence is indicated as either severe or yes (purple), no (yellow), or unknown (white). Symptoms are clustered by similarity and clustering is indicated by the dendrogram (left). Rows above the heatmap show metadata about each participant. Test results are indicated by color as either positive (black) or negative (light grey). Each participant's calculated probability of COVID-19 (P(COVID)) is indicated with higher probabilities shown in purple and lower probabilities in yellow. Participant sex, age, and cohort are also indicated. B) Box plots showing average cycle threshold (Ct) values (mean of N1 and N2 primers) for Cohort 1B (n = 19) for nasopharyngeal samples (NP), nasal samples, and saliva samples. Groups are compared using Kruskal-Wallis test by ranks. C) Box plots showing average Ct values (mean N1 and N2 primers) for Cohort 1B for each platform used. For all plots black circles are samples with concordance between nasal/saliva samples and NP samples (positive/positive or negative/negative). Grey squares show discordance between nasal/saliva samples and NP samples (positive/negative).

Supplemental Table 1: Contingency comparisons for Cohort 1A.

Cohort 1A	NP		
Nasal	Positive	Negative	Total
Positive	14	0	14
Negative	2	25	27

PPA	OPA
87.50%	95.12%

Cohort 1A	NP		
Saliva	Positive	Negative	Total
Positive	14	0	14
Negative	2	25	27

PPA	OPA
87.50%	95.12%

Cohort 1A	Nasal		
Saliva	Positive	Negative	Total
Positive	14	0	14
Negative	0	27	27

PPA	OPA
100.00%	100.00%

Supplemental Table 2: Contingency comparisons for Cohort 2.

Cohort 2 (n=20)	NP		
Nasal	Positive	Negative	Total
Positive	11	0	11
Negative	5	4	9

PPA	OPA
68.75%	75.00%

Cohort 2 (n=20)	NP		
Saliva	Positive	Negative	Total
Positive	12	2	14
Negative	4	2	6

PPA	OPA
75.00%	70.00%

Cohort 2 (n=20)	Nasal		
Saliva	Positive	Negative	Total
Positive	9	5	14
Negative	2	4	6

PPA	OPA
81.82%	65.00%

Supplemental Table 3: Contingency comparisons for Cohort 1B.

Cohort 1B	NP		
Nasal	Positive	Negative	Total
Positive	8	0	8
Negative	6	5	11

PPA	OPA
57.14%	68.42%

Cohort 1B	NP		
Saliva	Positive	Negative	Total
Positive	8	0	8
Negative	6	5	11

PPA	OPA
57.14%	68.42%

Cohort 1B	Nasal		
Saliva	Positive	Negative	Total
Positive	7	1	8
Negative	1	10	11

PPA	OPA
87.50%	89.47%

PPA: Positive Percent Agreement; OPA: Overall Percent Agreement

Supplemental Figure 4: Saliva and nasal collection devices.

A

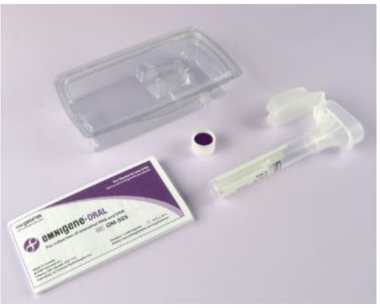

B

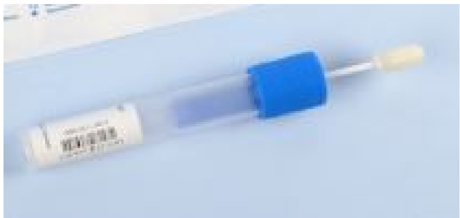

A) Saliva collection tube with funnel (OM-505). Patient was instructed to spit until saliva (not bubbles) reached the 1 ml fill-to-line before closing the funnel to release the stabilizing liquid. Patient removed the funnel before closing the tube with the provided purple lined cap. Patient was instructed to shake the tube for 10 seconds. B) Collection kit for nasal sample using blue cap tube containing swab (OCD-100). Patient was instructed to place swab sponge in one nostril and move it in a circular motion for 5 seconds before moving the sponge to the other nostril to repeat the circular motion for another 5 seconds. Without touching the sponge patients were instructed to unscrew the cap, insert the sponge into the tube, and close the cap tightly. With the tube capped, patients were instructed to shake the tube 15 times.

Clinical Quality Assurance Data.

The limit of detection of the CDC-based CLIA-LDT at our institution was validated between 560-1670 viral copies/mL depending on the specific viral extraction method used (4 distinct methods were validated to accommodate supply chain limitations; the Qiagen and Promega methods used on samples for this study were validated at 560 copies/mL). In comparison, the LOD for the commercial platforms were reported as 250 (Cepheid) and 500 (Diasorin) viral copies/mL in the respective EUA documents. Quality assurance (QA) data from our clinical laboratory shows the LDT has a PPA=90% (95% C.I. 74-96%) versus the Cepheid and a PPA=97% (95% C.I. 83-99%) vs. the Diasorin platforms in retrospective re-testing of archived samples on the LDT. All discordant samples in this QA data demonstrated late Ct values (>38) in the commercial test, consistent with variation around the limit of detection for these platforms. Furthermore, the LDT comparisons were inherently impaired, as all samples had been banked frozen and subjected to a freeze-thaw cycle prior to testing on the LDT. Parallel comparison was not possible due to multiple factors, including: severe supply chain limitations on the commercial platforms prohibiting non-essential testing, and physical separation of the commercial and LDT platforms across the health care system that prevented simultaneous processing. The level of inter-platform agreement we have observed is aligned with the ranges published by others[1-3].

Supplemental Table 4: Contingency comparison of CLIA-LDT and Cepheid.

		Cepheid				
		Positive	Negative			
CLIA-LDT	Positive	27	0		OPA	93%
	Negative	3	15		PPA	90%

Supplemental Table 5: Contingency comparison of CLIA-LDT and Diasorin.

		Diasorin				
		Positive	Negative			
CLIA-LDT	Positive	29	0		OPA	98%
	Negative	1	15		PPA	97%

References.

1. Zhen W, Manji R, Smith E, Berry GJ. Comparison of four molecular in vitro diagnostic assays for the detection of sars-cov-2 in nasopharyngeal specimens. J Clin Microbiol. 2020;58(8).
2. Procop GW, Brock JE, Reineks EZ, et al. A comparison of five sars-cov-2 molecular assays with clinical correlations. Am J Clin Pathol. 2021;155(1):69-78.
3. Lieberman JA, Pepper G, Naccache SN, Huang M-L, Jerome KR, Greninger AL. Comparison of commercially available and laboratory-developed assays for in vitro detection of sars-cov-2 in clinical laboratories. J Clin Microbiol. 2020;58(8).
